# South-West Orthopaedic Club

**Published:** 1972-10

**Authors:** 


### SOUTH-WEST ORTHOPAEDIC CLUB

A Meeting of the Club was held in Plymouth on the 29th April, 1972.

**SYMPOSIUM ON AMPUTATIONS**

**Amputations of the Upper Limb**—Dr. I. Fletcher (Roehampton).

**Present Problems in Limb Fitting**—Dr. C. E. Halliday (Limb Fitting Centre, Exeter and Plymouth).

**Future Trends in Limb Fitting**—Dr. R. G. Redhead (Roehampton).

**The Waterhouse-Friderichsen Syndrome and its Orthopaedic Consequences** — Surg. Commd. T. Hampton, R.N,. Surg. Capt. P. D. G. Pugh, R.N.

**Venous Gangrene**—Mr. N. Seymour (Plymouth)

**Surgical Treatment of Cervical Spondylosis**—Mr. W. E. Strachan (Plymouth) spoke of the treatment of neurological complications in cervical spondylosis by anterior cervical decompression and fusion. His comments were based upon a pilot study of 52 patients individually assessed in follow-up periods of 12 to 45 months. There were 33 males and 19 females, with a mean age of 56 years and an average duration of symptoms of 3 years. All patients had extensive conservative measures prior to surgery. Thirteen patients had pure radiculopathy, 11 myelopathy and 28 had a mixed picture; all were considerably incapacitated and 12 were either bedridden or barely ambulant. Radiological levels were determined by myelography and 79 levels were operated upon. The operation was the Harris modification of the Cloward technique. There were 26 single levels, 25 double and 1 had three levels operated on. Maximum lateral decompression of the nerve roots and radicular arteries was attempted and fusion was achieved with bone dowels obtained from the iliac crest. Of the patients under 60 years of age, 72% returned to their previous occupation, 16% obtained lighter jobs and 12% were not gainfully employed. The results were not influenced by age, sex or severity of symptoms nor the levels affected or treated. Five cases did not obtain material benefit from surgery and of these 4 had coexistent neurological disorders.

**Patellectomy for Chondromalacia Patellae**—Surg. Lt. Commd. A. C. Chakraverty, R.N., discussed the place of patellectomy in treatment of chondromalacia patellae. Chondromalacia is a particularly common problem in the Royal Navy. The overall results of patellectomy were good but they often tend to be disappointing in the adolescent. Undue prolongation of conservative treatment and multiple operations on the same knee were observed in the majority of the patients obtaining poor results after patellectomy. A moderate to extensive degree of new bone formation in the patellar bed after excision was seen in 54.5% of the patients who had been invalided from the Service after patellectomy. He suggested that further investigation was necessary into the problem of this new bone formation, which usually has an adverse effect on the outcome of the operation. The initial results of patelloplasty were encouraging and this procedure might well prove to be a better operation for chondromalacia, particularly for the younger age group and for females where a good cosmetic result is more desirable.

**The Sliding Nail for Intracapsular Fractures of the Femoral Neck**—Dr. S. K. Mukerjea (Plymouth) argued against the increasing tendency to carry out replacement of the femoral head for intracapsular fractures of the neck of the femur. He had been able to obtain good early results using a sliding nailplate. He stressed the need to obtain a good reduction with the head in slight valgus, and acurate internal fixation was essential.

**An Operation for Dupuytren's Contracture**—Dr. J. G. Hazelwood (Plymouth) described a technique for Dupuytren's Contracture involving two or more fingers. A palmar V incision, one limb extending to the ulnar border of the little finger the other to the web space of the index and middle fingers, is made. The skin flap, and where possible the subcutaneous fibro-fatty layer, is reflected distally. This gives a good exposure allowing a radical fasciectomy, avoiding damage to the neuro-vascular bundles, to be done under direct vision. Contractures of the metacarpal-phalangeal joints can be dealt with but proximal interphalangeal joint contractures may require a separate Z-plasty. Skin closure is obtained by converting the V into a Y plasty. There were no complications in 19 cases except sloughing of the skin flap in one patient who had had two previous operations on the hand.

**Reflections on Six Years' Experience of A.O. Techniques**—Surg. Capt. P. D. G. Pugh, R.N.

**Some Lesions of the Clavicle**—Mr. F. T. Wheeldon (Plymouth) gave an account of 4 patients with unusual problems involving the clavicle. One patient, who had had a fracture dislocation of the sterno-clavicular joint internally fixed with a Kirschner wire, developed an unusual chest complication due to migration of the Kirschner wire down into the chest and into the pericardium.

**The Pelvic Aspect of the Acetabulum in Charnley's Arthroplasty**—Mr. N. Seymour (Plymouth). Following a case of thrombosis of the external iliac vein and a case of spasm of the pelvic colon after Charnley's arthroplasty, the acetabulum was studied. In a number of post mortem dissections the floor of the acetabulum was drilled and it was found that the pelvis was penetrated between the internal and external iliac veins and always very close to the obturator nerve. The anatomy varied so that sometimes either vein was close to the drill point. Also the thickness of the acetabular floor and the bulk of the obturator internus muscle varied considerably. In order to study the temperature reached in the vicinity of tissues deep to the floor of the acetabulum, some Charnley arthroplasties were performed using a posterior approach. The drill hole was palpated from the sciatic notch and a thermocouple placed on the pelvic side of obturator internus in the vicinity of the hole. Measurements have to date been carried out in eight patients, some with and some without the use of the stainless steel restrainer. The rise in temperature after insertion of the cement is small, being a maximum of 3°C without the restrainer and with an average rise of only $\frac{1}{2}$°C when the restrainer is used.

**Nelson, His Wounds and His Ceramics**—Surg. Capt. P. D. G. Pugh, R.N.

